# Cultural Competence in Pediatrics: Health Care Provider Knowledge, Awareness, and Skills

**DOI:** 10.3390/ijerph13010014

**Published:** 2015-12-22

**Authors:** Kirk Dabney, Lavisha McClarin, Emily Romano, Diane Fitzgerald, Lynn Bayne, Patricia Oceanic, Arie L. Nettles, Laurens Holmes

**Affiliations:** 1 Nemours Office of Health Equity and Inclusion, Wilmington, DE 19803, USA; kirk.dabney@nemours.org (K.D.); lavisha.mcclarin@hotmail.com (L.M.); ejr9bs@virginia.edu (E.R.); patricia.oceanic@nemours.org (P.O.); 2 Epidemiology and Biostatistics Department, University of Maryland-College Park, College Park, MD 20742, USA; 3 College and Graduate School of Arts & Sciences, University of Virginia, Charlottesville, VA 22904, USA; 4 Nursing Department, Nemours/A. I. DuPont Hospital for Children, Wilmington, DE 19803, USA; diane.fitzgerald@nemours.org (D.F.); lynn.bayne@nemours.org (L.B.); 5 Office of Inclusion and Health Equity, Monroe Carell Jr. Children’s Hospital at Vanderbilt, Nashville, TN 37232, USA; arie.l.nettles@vanderbilt.edu; 6 Biological Sciences Department, University of Delaware, Newark, DE 19716, USA

**Keywords:** cultural competence, culturally competent care, cultural diversity, training program, health personnel, pediatrics

## Abstract

The purpose of this study was to assess the effects of a cultural competence training (CCT) program on pediatric health care providers’ self-reported ability to provide culturally competent care to a diverse pediatric patient population. This quantitative, nested ecologic level study design used a repeated measure in the form of pre-test and post-test data to assess percent change in providers’ cultural awareness, experience working or learning about different cultures, and preparedness and skills in working with different cultures before and after CCT. The study was conducted between 2011 and 2012 in a pediatric hospital and associated outpatient offices. The sample consisted of pediatric health care providers from various departments, mainly physicians and nurses (*n* = 69). Participants completed a pre-intervention cultural competence assessment and then were subjected to a cultural competence-training program, after which they completed the assessment a second time. The baseline and post-intervention data were collected in the form of Likert scales and transformed into a quintile or quartile scale as appropriate. Data were assessed using paired *t*-tests or Wilcoxon’s signed-rank tests. Providers indicated a 13% increase in knowledge (53.9% *vs.* 66.7%, *t* = 3.4, *p* = 0.001), 8.7% increase in awareness (46.7% *vs.* 55.4%, *t* = 3.0, *p* = 0.002), and 8% statistically marginal increase in skills (66.4% *vs.* 74.5%, *z* = 1.8, *p* = 0.06). Culturally competent training in a pediatric environment significantly enhances knowledge, awareness and to some extent skills in providing care to culturally diverse patient population.

## 1. Introduction

There is a growing awareness in the fields of public health and medicine that different populations of people can have different health care experiences and outcomes [1,2]. Health disparities can refer to any difference in health between groups, but the 2002 landmark report *Unequal Treatment* identified racial and ethnic minorities as being particularly at risk for receiving a lower quality of care when compared with non-minorities [2]. The causes of health and health care disparities are complex and include the social determinants of health, structural barriers at the health care system level, leadership/workforce barriers, and patient/provider communication barriers. The need to address racial and ethnic health disparities is becoming more urgent, as the United States population continues to diversify. According to the 2014 National Projections by the U.S. Census Bureau, 50.2% of American children under the age of 18 years will belong to a racial or ethnic minority group by the year 2020 [3].

There have been numerous strategies proposed to reduce health disparities that have been aimed at some of the causes outlined above [4]. Cultural competence training (CCT) for health care providers is one strategy. Culture is a complex concept, but it can be broadly defined as a person’s way of life or association with various population groups, including a person’s racial, ethnic, social, economic, geographic, and/or linguistic attributes or identities [5]. Cultural competence training aims to improve patient-provider communication by doing the following: (1) assisting health care providers in recognizing cultural diversity and the role of culture in shaping their patients’ health beliefs, behavior, and outcomes; (2) improving provider understanding of health disparities and their complex roots; and (3) teaching providers how to practice patient-centered, culturally appropriate care for each unique patient [6,7]. Ultimately, CCT aims to make health care a more inclusive environment for patients of all cultural backgrounds at its most basic level: the patient-provider interaction [6].

Current evidence suggests that CCT can improve attitudes, knowledge, and/or skills of pre-professional and professional health care providers, although there is great diversity across these training programs in terms of duration, content, specific aims, and evaluation measures [8,9,10,11,12]. When compared to the amount of literature investigating the effect of CCT for pre-professional or adult providers, there are fewer data that demonstrate its effect specifically in pediatric environments [13]. The purpose of this study was to assess health care providers’ ability to provide culturally competent care in both inpatient and outpatient pediatric settings before and after completion of a CCT program. We aimed to identify the effects of the CCT program on providers’ awareness of and sensitivity towards patients’ cultural differences and their knowledge/skills with regard to providing effective care for patients and families of diverse cultural backgrounds.

## 2. Experimental Section

### 2.1. Materials and Methods

Following Institutional Review Board approval, we performed a nested ecologic level repeated measure design to determine the effects of CCT on providers’ cultural awareness/sensitivity, experience working with or learning about diverse cultures, and preparedness and skills in providing culturally competent care. This design is a prospective within-subject study that uses cases as their own controls, thereby minimizing confounding.

#### 2.1.1. Sampling

The study sample included 69 pediatric health care providers. Participants were sampled from a pool of associates employed at Nemours Children’s Health System (NCHS) in 1 January 2011 and 31 December 2012. Due to loss to follow-up, the post sample subjects represented an estimated 89.6% of the baseline sample (*n* = 77). The employees were selected from several NCHS units and facilities: rehabilitation inpatient unit, surgery/surgical subspecialty inpatient unit, hematology/oncology outpatient unit, emergency department, Dover outpatient office, and Nemours St. Francis outpatient facility.

#### 2.1.2. Questionnaire/Instrument

All participants completed a pre-test consisting of 23 questions, each corresponding to a Likert scale, to assess participants’ baseline cultural competency. Following the test, all participants completed a two-part CCT program consisting of an independently completed web-based portion (approximately 2.5 h) and a facilitated group discussion (approximately 1.5 h). The web-based training was tailored to each participant’s specific position at NCHS (nurse, physician, case manager, or non-clinical employee). Upon the completion of the web-based training, the system tracked completed and uncompleted status of participants which allowed us to send e-mail to department chairs to encourage course completion. We assessed those who did not complete (*n* = 9) the post intervention assessment and observed no significant variability by sex, age group, race and professional level between those who did and did not complete the posttest. Both the pre- and post-test were administered electronically which minimizes socially acceptable response bias associated with researcher-assisted administration, thus increasing the internal validly of the assessment.

Both portions of the CCT were based on the quality interactions cross-cultural framework, which identifies five key aspects of a “quality interaction” between patient and provider: assessing cross-cultural issues, addressing language and literacy, exploring illness/treatment beliefs, determining social context, and engaging in negotiation [14]. Further detail on the training is available elsewhere [14]. Approximately one year post training, participants completed a post-test that consisted of the same 23 questions as in the baseline assessment.

#### 2.1.3. Power and Sample Size Estimations

To estimate the power and sample size, we used a Type 1 error tolerance of 5% (0.05), Type 2 error tolerance (β) of 20% (0.2), and effect size of 0.1. With these parameters, we determined the sample size to be 66. Consequently, with this effect size (Δ), the study is able to detect a difference of 10% when comparing baseline and post-intervention data.

### 2.2. Statistical Analysis

The data from the pre-tests and post-tests were collected in the form of self-reported scores on 4- or 5-point Likert scales. Prior to statistical analysis, the 5-point Likert scale was transformed into a quintile scale, and the 4-point Likert scale was transformed into a quartile scale according to the schema presented in Table 1.

**Table 1:** Likert score transformation into quintiles or quartiles ^a^.

Question Group	Likert Scale	Quintiles/Quartiles
Questions 1–9	1—strongly disagree	20
	2—disagree	40
	3—neither agree or disagree	60
	4—agree	80
	5—strongly agree	100
Questions 10–14	1—no instruction at all	25
	2—little	50
	3—a comfortable amount	75
	4—a great amount	100
Questions 15–19	1—very unprepared	20
	2—somewhat unprepared	40
	3—somewhat prepared	60
	4—well prepared	80
	5—very well prepared	100
Questions 20–23	1—not at all skillful	25
	2—somewhat unskillful	50
	3—somewhat skillful	75
	4—very skillful	100

^a^ Individual scores were transformed into quartile or quintile scores prior to the computation of the mean scores presented in Table 2, Table 3, Table 4 and Table 5.

This ordinal data transformation technique is validated by the central tendency theorem, which maintains that quantitative data tends to congregate around some central value [15]. In this case, the mean value for Likert scale data tends to remain consistent across various scale forms; from which mean percentage scores were calculated (Table 2, Table 3, Table 4 and Table 5). This technique of transforming quartile or quintile scale data into percentages has also been validated in previous studies [16,17,18]. We transformed the Likert scale data to achieve near normality in the shape and distribution of the data, which is required for meaningful parametric analysis (such as *t*-test or ANOVA). When the normality assumption was violated, a non-parametric equivalent test to correlated or paired *t*-test was used.

**Table 2:** Providers’ cultural awareness and sensitivity survey results ^a,b^.

Question: Rate the Following with Respect to Your Overall Awareness and Sensitivity to Cultural Differences:	Pre-Test Mean	Pre-Test SD	Post-Test Mean	Post-Test SD	*t*	*z*	*p*
(Q1) I feel that when I have an assignment that includes a culturally diverse patient that I spend less time with that patient than my other patients	41.3	17.87	38.55	19.8	1.04	-	0.15
(Q2) I treat all patients, families, and co-workers with respect for their culture, even though it may be different from mine	86.49	22.35	92.75	9.68	-	1.38	0.17
(Q3) I am aware that the roles family members play may differ between or by culture	88.57	18.19	92.17	9.83	-	1.14	0.25
(Q4) I understand how culture can impact childrearing practices	85.19	18.75	88.99	13.08	-	0.76	0.45
(Q5) I have no problems accepting and providing services to parents who are LGBT (Gay, Bisexual, Lesbian, Transgender)	90.13	18.24	91.88	12.52	-	0.62	0.54
(Q6) Sometimes I unconsciously impose my beliefs and value systems onto my patients, families, and coworkers	46.67	23.49	38.84	17.45	-	2.12	0.03
(Q7) Sometimes I unconsciously participate in insensitive comments or behaviors towards patients, families, or co-workers with different cultural beliefs other than my own	40.58	17.77	35.65	17.11	-	2.05	0.04
(Q8) In general, the U.S. healthcare system treats all patients equally regardless of cultural differences that may exist	52.73	22.92	50.72	18.65	0.08	-	0.94
(Q9) I feel that when I have an assignment that includes a culturally diverse patient that I spend more time with that patient than my other patients	46.67	16.47	55.36	19.14	2.99	-	0.001

Notes: ^a^ pre-test mean/post-test mean are presented in percentages transformed from Likert scale values; ^b^ *t* value presented for normally distributed data, *z* value presented for non-normally distributed data.

**Table 3:** Training and experience ^a,b^.

Question: How Much Instruction have You Received Regarding the Following Cross-Cultural Aspects:	Pre-Test Mean	Pre-Test SD	Post-Test Mean	Post-Test SD	*t*	*z*	*p*
(Q10) Determining how patients or families from different cultures want to be addressed	53.99	21.28	66.67	19.01	3.42	-	0.001
(Q11) Assessing the patient or family understanding of the cause of the patient’s illness	56.52	22.55	62.68	19.47	1.70	-	0.04
(Q12) Identifying how well a patient or family can read or write English	53.26	21.83	59.06	21.85	1.49	-	0.07
(Q13) Identifying cultural (religious and non-religious) customs that might affect care	53.99	20.84	63.77	18.46	2.95	-	0.002
(Q14) Delivering services effectively through an interpreter	67.39	20.7	67.75	17.73	-	0.33	0.74

Notes: ^a^ pre-test mean/post-test mean are presented in percentages transformed from Likert scale values; ^b^ *t* value presented for normally distributed data, *z* value presented for non-normally distributed data.

**Table 4:** Provider preparedness ^a,b^.

Question: How Prepared do You Feel You are to Care for Patients or Families:	Pre-Test Mean	Pre-Test SD	Post-Test Mean	Post-Test SD	*t*	*z*	*p*
(Q15) with limited English proficiency	62.61	19.38	70.14	18.98	-	1.84	0.065
(Q16) from cultures different from your own	66.38	15.9	74.49	14.09	-	2.74	0.006
(Q17) whose religious beliefs affect treatment	62.61	19.07	70.43	14.8	-	2.28	0.02
(Q18) who are members of racial and ethnic minority populations	71.59	18.91	76.52	18.77	-	1.94	0.05
(Q19) with health beliefs or practices different from your own	66.09	18.57	74.49	14.91	-	2.59	0.01

Notes: ^a^ pre-test mean/post-test mean are presented in percentages transformed from Likert scale values; ^b^ *t* value presented for normally distributed data, *z* value presented for non-normally distributed data.

**Table 5:** Provider skills ^a,b^.

Question: How Skillful do You Feel You Are at the Following:	Pre-Test Mean	Pre-Test SD	Post-Test Mean	Post-Test SD	*t*	*z*	*p*
(Q20) Identifying how well a patient can read or write English	61.23	20.8	66.67	18.5	-	1.21	0.22
(Q21) Identifying religious beliefs that might affect clinical care	60.14	21.57	63.77	16.9	-	0.61	0.54
(Q22) Identifying cultural customs that might affect clinical care	58.33	20.86	65.58	16.1	-	1.85	0.06
(Q23) Working effectively through a medical interpreter	75	19.17	75.36	17.4	-	0.19	0.84

Notes: ^a^ pre-test mean/post-test mean are presented in percentages transformed from Likert scale values; ^b^ *t* value presented for normally distributed data, *z* value presented for non-normally distributed data.

Prior to hypothesis-driven analysis, data were screened for missing variables and outliers. The summary statistics for categorical variables, such as sex, were estimated using frequency and percentages. Continuous variables were assessed for normality distribution prior to computation of the summary statistics. The normally distributed data were examined using mean and standard deviation while the data that violated the normality assumption were summarized using median and interquartile range.

We hypothesized that CCT improves participants’ cultural awareness, knowledge, skills, experience, and preparedness to provide culturally competent care. To test this hypothesis, we reexamined the data to determine which questions on the baseline and post-test assessment were normally distributed. All normally distributed data were assessed using a paired *t*-test, while non-normally distributed data were assessed using Wilcoxon’s signed-rank test, a non-parametric equivalent for correlated *t*-test. STATA fits the Wilcoxon’s signed-rank test using: *signed rank variable 1[pre-test question 1] = variable 1[post-test question 1].* The type 1 error tolerance was set at 0.05 (5%). Some questions were two-tailed and others were one-tailed, depending on the nature and direction of the question. For example, if a question implied a specific directionality (e.g., an increase or decrease) a one-tailed test was used, but if it was bidirectional a two-tailed test was used. All data were analyzed using STATA statistical software, version 13.0 (STATACorp., College Station, TX, USA).

## 3. Results and Discussion

### 3.1. Results

The assessment of demographic data indicated blacks/African Americans (*n* = 7, 10.14%), whites/Caucasians (*n* = 49, 71.01%), and others (*n* = 13, 18.85%). The majority of the participants were female providers (*n* = 56, 81.16%), and the pediatric health care providers were nurses, physicians, physical therapists, *etc.*).

#### 3.1.1. Providers’ Cultural Awareness and Sensitivity

Table 2 illustrates the effects of CCT on providers’ cultural awareness and sensitivity. There were nine questions within this construct; of these, three indicated statistically significant finding. With respect to the question, *sometimes I unconsciously impose my beliefs onto my patients, families and coworkers*, there was a significant 8% decrease in that belief (46.7% *vs.* 38.8% comparing the pre- and post-tests, respectively, *z* = 2.1, *p* = 0.03). Regarding the question, *sometimes I unconsciously participate in insensitive comments or behaviors towards patients, families or coworkers with different cultural beliefs other than my own*, there was a significant 5% decrease in that practice (40.6% *vs.* 35.7% comparing the pre- and post-tests, *z* = 2.05, *p* = 0.04). With respect to the question, *I feel that when I have an assignment that includes a culturally diverse patient that I spend more time with that patient then with other patients,* there was a significant 8.7% increase in time spent with culturally diverse patients, (46.7% *vs.* 55.4% comparing pre- and post-test scores, *t* = 3.0, *p* = 0.002).

#### 3.1.2. Education and/or Experience

Table 3 illustrates the level of education and/or experience in various areas of cultural competence. Concerning the question, *determining how patients or families from different cultures want to be addressed or interacted with*, there was a significant 13% increase in the ability of the health care provider to make this determination (53.9% *vs.* 66.7%) comparing the pre- and post-tests, *t* = 3.4, *p* = 0.001. With respect to the question, *assessing the patient or family understanding of the course of the patient’s illness*, there was a significant 6% increase in the ability to perform this task (56.5% *vs.* 62.7%, *t* = 1.70, *p* = 0.04). With regard to *identifying how well a patient or family can read or write English* (patient’s linguistic capacity), there was a statistically marginally significant 6% increase in providers’ ability (53.3% *vs.* 59.1% comparing the pre- and post-tests, *t* = 1.50, *p* = 0.07). With respect *to identifying cultural (religious and non-religious) customs that might affect care*, there was a significant 10% increase in providers’ ability (53.9% *vs.* 63.8% comparing the pre- and post-tests, *t* = 3.0, *p* = 0.002). Regarding *delivering services effectively through a medical interpreter*, there was no statistically significant difference in the response comparing the baseline with post intervention score (*z* = 0.33, *p* = 0.74).

#### 3.1.3. Provider Preparedness

Table 4 presents the providers’ preparedness to care for culturally diverse patients or families. Regarding *how prepared providers are to care for patients or families with limited English proficiency*, there was a statistically marginally significant 8% increase in preparedness (62.6% *vs.* 70.1% comparing pre- and post-tests, *z* = 1.8, *p* = 0.06). Concerning *how prepared providers are to care for patients or families from cultures different from their own*, there was an 8% increase in this ability (66.4% *vs.* 74.5% comparing pre- and post-tests, *z* = 2.74, *p* = 0.01). With respect to *the provider’s preparedness to care for patients whose religious beliefs affect treatment*, there was a significant 8.2% increase in preparedness (62.6% *vs.* 70.4% comparing pre- and post-tests, *z* = 2.3, *p* = 0.02). With regard to *the provider’s ability or preparedness to care for patients who are members of a racial and ethnic minority* (disparity populations), there was a 5% increase in preparedness but it was only statistically marginally significant (71.6% *vs.* 76.5% comparing the pre- and post-tests, *z* = 1.94, *p* = 0.05). Concerning *how providers are prepared to deal with health beliefs or practices that are different from their own*, there was a significant 8% increase in preparedness (66.1% *vs.* 74.5% comparing the pre- and post-tests, *z* = 2.6, *p* = 0.01).

#### 3.1.4. Provider Skills

Table 5 illustrates providers’ skills in providing culturally competent care. With respect *to identifying how well a patient can read or write English*, there was a statistically non-significant 5% increase in perceived skill (61.2% *vs.* 66.7% comparing pre- and post-tests, *z* = 1.2, *p* = 0.2). Concerning *identifying religious beliefs that might affect clinical care*, there was a statistically nonsignificant 3% increase in perceived skill (60.14% *vs.* 63.8% comparing pre- and post-tests, *z* = 0.6, *p* = 0.54). With respect to *identifying cultural customs that might affect clinical care*, there was a statistically marginally significant 7.5% increase in perceived skill (58.3% *vs.* 65.6% comparing pre- and post-tests, *z* = 1.85, *p* = 0.06). When it comes to *working effectively through a medical interpreter*, providers showed no difference in perceived skill improvement following CCT (*z* = 0.1, *p* = 0.84).

### 3.2. Discussion

This study was proposed to assess the effects of CCT on pediatric health care providers’ knowledge, awareness, and skills in providing health care across a diverse pediatric inpatient and outpatient population. We used a repeated measure design and obtained some relevant findings. First, CCT improved providers’ cultural awareness and sensitivity to patients’/families’ cultural needs. Second, CCT improved providers’ knowledge of what constitutes culturally competent care and what steps should be taken to ensure effective practice. Third, CCT enhanced providers’ preparedness and comfort level in caring for patients with various culturally diverse attributes. Finally, CCT to some extent improved providers’ skills in practicing culturally competent care, specifically identification of patient/family language proficiency and pertinent cultural customs.

We have demonstrated that administering CCT to health care providers, specifically nurses and physicians, improves their cultural awareness and sensitivity. Our multifaceted training program, web-based training which included didactics, case studies, and facilitated discussion, allowed participants to learn about and reflect upon the complexities of multicultural interactions. According to participants’ self-assessments, they felt more aware of and sensitive towards cultural differences following CCT. While our finding in this dimension is supported by previous studies [8,9,10], a study observed no effect of CCT on child health nurses’ cultural awareness [12].

Our findings suggest that CCT can improve providers’ knowledge of the core constituents of culturally competent care. This finding is indicative of the potential of CCT in enhancing the ability to provide culturally and linguistically appropriate care for each patient as defined by the Culturally and Linguistically Appropriate Standards (CLAS) set forth by the U.S. Department of Health and Human Services [19]. Following training, our providers were more cognizant of how to interact with patients/families, assess patient/family understanding of their health situation, how to identify patient/family language proficiency, and identify pertinent cultural and/or spiritual/religious beliefs of patients/families. Again, our data are supported by previous studies that demonstrated the effect of CCT on provider knowledge and/or understanding of culturally competent care [8,9,12].

We have illustrated how providers’ involvement in training increased their preparedness and confidence level in caring for patients from diverse cultural backgrounds. Following exposure to specific information regarding differences in language proficiency; various religious affiliations; the complexities of concepts such as culture, race, and ethnicity; and differing health belief models, providers felt more prepared to care for a diverse set of patients. There is opportunity for further research into the degree to which providers were able to work across cultures in a humble and self-reflective manner, recognizing their own biases and how they affect their interactions with patients. This element of self-evaluation has been identified as a key component of multicultural education [20,21].

Our data to some extent indicate improved provider skills following CCT. Although imprecise, we observed clinically reasonable differences in skills following CCT. Specifically, providers felt more able to identify patient/family cultural and linguistic attributes that could affect the patients’ health and health care. The increase in skill level observed in these dada could be due to the fact that our training program was results-driven and evidence-based and sought to provide participants with information relevant to their day-to-day practice [14]. Our findings are supported by other studies that found increases in skills following CCT [8,11,12]. However, other CCT programs evaluated in the literature differ substantially with respect to content of materials, knowledge of facilitators, duration and number of trainings, level of interaction, and training environment [8,9,10,11,12]. Further, the duration of our CCT which is relatively longer than average in literature may serve as enhancer of skills in providing a culturally competent care to culturally diverse pediatric patients and families.

Overall, these data indicate that CCT improved providers’ knowledge, awareness, and to some extent skills with respect to cultural awareness and sensitivity, increasing training and experience in working with culturally diverse patients, improving providers’ preparedness to work with these patients, and improving providers’ practical skills in providing culturally competent care.

Despite the strengths of this study, namely, rigorous CCT intervention design and relatively adequate sample size, there are some limitations. First, the sampling scheme appeared to be inadequate given that an ecologic level design was used. While our post-intervention (post-test) sample reflected an estimated 80% of the pre-intervention (pre-test) sample, we performed our analysis using a repeated measure. This approach tends to introduce non-differential misclassification bias. However, we do not surmise that our post intervention data are driven solely by this non-differential misclassification bias. Finally, while our study findings illustrate the beneficial effects of CCT in improving providers’ self-reported ability to practice culturally competent care, there is a possibility of contamination from other sources (conferences, seminars, continuing medical education, *etc.*) that could also explain in part the substantial knowledge, awareness, and skills gained following our training [22].

## 4. Conclusions

In summary, CCT in a pediatric environment has the potential to improve knowledge, awareness, and to some extent skills of health care providers and thereby improve their ability to provide culturally competent care. Consequently, CCT illustrates the ability to promote health equity, leading to improvements in the six Institute of Medicine quality pillars (safety, effectiveness, timeliness, patient-centeredness, and equity). Additionally, this study is suggestive of the need to initiate and sustain CCT on a routine basis as one of the essential vehicles in optimizing care across diverse pediatric patient populations. Given the variability in the assessment materials and the duration of training as main issues in determining the effect of CCT in improving patient care, we suggest large sample and sub-providers assessment with level-specific materials, 36 months intervention period with booster sessions, which is the future direction for our intervention mapping and implementation.
